# Early CRP kinetics predict surgical outcomes in patients with Hartmann reversal

**DOI:** 10.3389/fsurg.2026.1732382

**Published:** 2026-02-02

**Authors:** Bertha Dimas, Guillermo Enrique Hernández, Gisela Oropeza, Ivonne Peralta, Jeziel Ordoñez, Luis Enrique Bolaños, Agustín Güemes, Billy Jiménez, Mario Zambrano, Cittim B. Palomares-Palomares, Eduardo Rios-Garcia

**Affiliations:** 1 Facultad de Medicina, Universidad Nacional Autónoma de México (UNAM), Mexico, Mexico; 2 Colorectal Surgery Unit, Hospital General de México (HGM), Mexico, Mexico; 3 Surgery Unit, Hospital General de México (HGM), Mexico, Mexico; 4 Anesthesiology Unit, Centro Medico ABC, Mexico, Mexico; 5 Facultad de Ciencias Administrativas y Sociales, Universidad Autónoma de Baja California, Ensenada, Mexico; 6 Centro de Investigación en Ciencias de la Salud (CICSA), Facultad de Ciencias de la Salud, Universidad Anáhuac, México, Mexico

**Keywords:** CRP—C-reactive protein, CRP kinetics, Hartmann reversal, predictive test, surgical success

## Abstract

**Background:**

Hartmann reversal (HR) is associated with considerable morbidity, and early identification of patients at risk of complications is crucial. C-reactive protein (CRP) is a well-established inflammatory biomarker, but its dynamic changes (ΔCRP) as a predictor of surgical success in HR remain unclear. This study evaluates the role of ΔCRP between postoperative days 1 and 3 in predicting surgical outcomes.

**Methods:**

A retrospective observational study was conducted on patients undergoing HR at a tertiary hospital between January 2023 and December 2024. Demographic, clinical, and surgical data were collected. ΔCRP was defined as the absolute difference between CRP levels on postoperative days 1 and 3. Surgical success was defined as the absence of major complications, no need for reoperation, and discharge within the expected recovery period. Statistical analyses included logistic regression and receiver operating characteristic (ROC) curve analysis to determine the predictive value of ΔCRP.

**Results:**

A total of 83 patients were included. ΔCRP was significantly higher in patients with complications (56 vs. 136 mg/L, *p* < 0.001). ROC analysis identified an optimal ΔCRP cutoff of 113.1 mg/L for predicting surgical success (AUC = 0.754). Logistic regression confirmed ΔCRP as an independent predictor of success (OR: 1.015, 95% CI: 1.010–1.020, *p* < 0.001).

**Conclusions:**

ΔCRP is a valuable predictor of surgical success in HR. Patients with persistently elevated CRP levels may benefit from closer monitoring and early interventions. Integrating CRP kinetics into postoperative protocols could optimize patient outcomes and resource allocation.

## Introduction

Hartmann procedure is widely used to manage complicated colorectal disease that includes perforated diverticulitis, malignancy, and obstruction (1). The surgery, often lifesaving, It leaves an end colostomy that later needs restoration of continuity through Hartmann reversal (HR) (2). Living with a stoma brings functional limits and psychosocial burden that many patients wish to resolve. HR is a demanding procedure performed in patients who often have comorbidity. Reported morbidity includes anastomotic leakage, infectious complications, ileus, and prolonged hospitalization (3–5). Teams need early and objective signals to guide selection, postoperative surveillance, and timely rescue. These signals can help reduce preventable harm and focus resources where they matter most (6).

**Figure 1:**
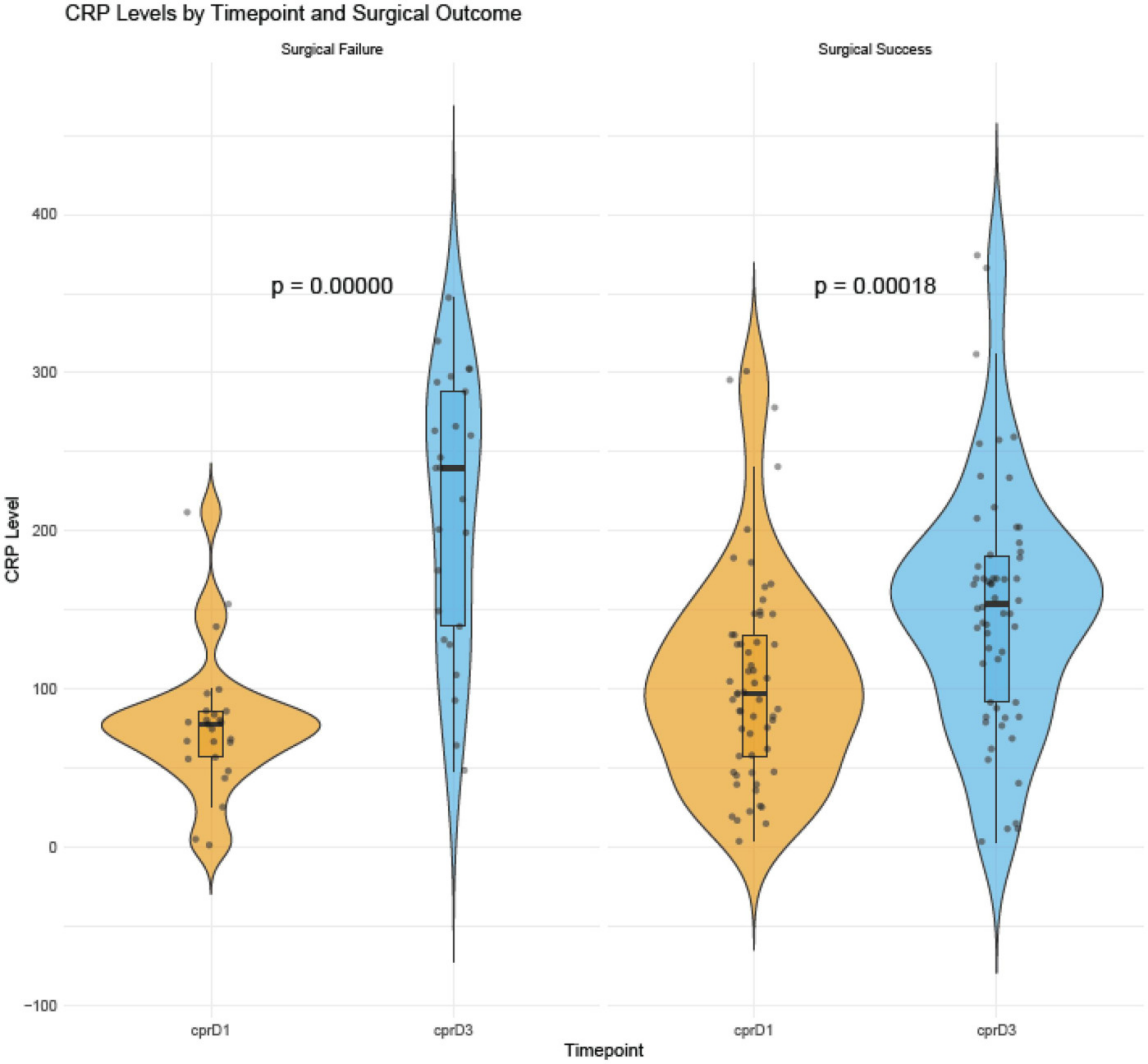
CRP levels by timepoint and surgical outcome. Violin plots show the distribution of C-reactive protein (CRP) levels at Day 1 (crpD1) and Day 3 (crpD3). p-values displayed within each panel correspond to the within-outcome comparison between Day 1 and Day 3.

C reactive protein (CRP) is a well established indicator of systemic inflammation. It rises after tissue injury and infection and usually falls as recovery proceeds (7, 8). In abdominal and colorectal surgery, higher early postoperative CRP has been linked with anastomotic leakage and surgical site infection (9, 10). Many pathways therefore include CRP on the first and third postoperative days. Single measurements can inform decisions, yet they are sensitive to sampling time, fluid shifts, analgesic regimens, and antibiotics. Clinicians benefit from measures that track the trajectory of healing rather than one isolated point. Such measures can align laboratory data with bedside assessment and support consistent actions across the team.

Recent work has shifted attention from absolute CRP values to CRP kinetics as candidate predictors of outcome (9, 11, 12). Evidence specific to HR is still limited, and external validation is scarce. Across colorectal and other major abdominal procedures, consensus on thresholds and optimal timing has not been reached (13–15). This window often captures the early inflammatory peak and the expected decline in uncomplicated courses (9, 11, 12). Failure of CRP to fall during this period may signal ongoing inflammation, infection, or an occult leak. A clear decline is more consistent with stable recovery (13–15).

The present study evaluates whether the change in CRP between postoperative day 1 and day 3, expressed as ΔCRP, predicts surgical success after HR. We aim is to test if an ΔCRP identifies patients who recover without major complications and leave hospital within the expected period. This could offer a simple, but reproducible marker that supports early risk stratification and day to day decisions in routine postoperative care.

## Methods and patients

### Study design

We performed a retrospective observational study at a third level public hospital. The study window was January 2023 to December 2024. The institutional review board approved the protocol. Because of the retrospective design, informed consent was waived. All data were anonymized before analysis. The study reflected routine clinical practice and did not alter patient management.

### Patients

Eligible patients had a colostomy suring Hartmann procedure with adequate conditions for reconnection and complete clinical and biochemical information. We included consecutive cases within the study window. Patients were identified through operative logs and electronic records. Exclusion criteria were incomplete medical CRP values on both postoperative day 1 (POD1) and postoperative day 3 (POD3), active malignancy, chronic immunosuppressive therapy (including long-term systemic corticosteroids or other immunomodulators), or a concurrent infectious process unrelated to surgery. These criteria were chosen to reduce baseline inflammatory noise and isolate surgery-related changes in CRP; the final cohort represents all cases that met these criteria during the period.

### Outcomes definition

The main exposure was the change in CRP between POD1 and POD3. We expressed this as ΔCRP, defined as the absolute difference between CRP values measured on postoperative day (POD) 1 and POD3, and we included only patients with complete records containing both measurements, which are routinely obtained at our institution. Sampling times were not protocolized to a fixed clock time and corresponded to the CRP determinations documented for each respective postoperative day in routine care; because all assessed patients fulfilled the completeness criterion, no imputation for ΔCRP was required. Surgical success was defined *a priori* as an uncomplicated postoperative course, operationalized as absence of major complications, no unplanned reoperation within 30 days, and discharge within the expected postoperative length of stay for Hartmann reversal (≤7 postoperative days at our institution). Major complications were defined as postoperative events requiring surgical, endoscopic, or radiologic intervention, admission to the intensive care unit, or resulting in organ failure or death; prespecified events included anastomotic leak, prolonged ileus requiring nasogastric decompression beyond postoperative day 5, intra-abdominal abscess, and deep or organ-space surgical site infection. We did not formally grade complications using the Clavien–Dindo classification, but used this pragmatic definition based on intervention and level of care, which reflects routine decision-making after Hartmann reversal. Surgical failure (SF) was defined as the presence of any complication during that period. These definitions align with routine decision making after HR.

### Data collection

We abstracted age, sex, and body mass index from the electronic medical record. Preoperative laboratory variables included hemoglobin, hematocrit, leukocytes, platelets, and albumin. Surgical variables included indication for HR, operative approach, duration of surgery, and estimated blood loss. Postoperative CRP was recorded on POD1 and POD3. Data abstraction followed a standardized template to maintain consistency filled by an independent blinded team. When a data field was unclear, the source note in the record was reviewed to confirm the value.

### Sample size and statistical analysis

This observational cohort included all eligible cases in the study period. With 83 patients and ∼30% SF, there were approximately 25 events, permitting adequate logistic regression. *post hoc* precision analysis indicates that, for an area under the receiver operating characteristic (ROC) curve (AUC) near 0.75, adequate to judge discriminative value and motivate prospective validation.

We also used ROC analysis to estimate the discriminative performance of ΔCRP and to identify an optimal cutoff based on Youden's Index. To assess internal validity, we performed bootstrap resampling with 2,000 iterations, recalculating the AUC and Youden's optimal cut-off in each resample. We summarized the bootstrap distribution using mean and percentile-based 95% confidence intervals (CI). In addition, we conducted a sensitivity analysis by evaluating the performance of nearby ΔCRP thresholds (±5%–10% around the optimal cut-off), reporting sensitivity, specificity, PPV, NPV and confusion-matrix counts for each threshold.

Continuous variables were summarized as means with standard deviations or as medians with interquartile ranges, according to distribution. Categorical variables were reported as counts and percentages. Comparisons between surgical success and surgical failure used Student *t*-test or Mann–Whitney *U* test for continuous data and chi square or Fisher exact test for categorical data. We fitted multivariable logistic regression models to evaluate the association between ΔCRP and surgical success. Candidate covariates included body mass index (BMI), surgical time, intraoperative bleeding, serum albumin, neutrophil-to-lymphocyte ratio (NLR), and platelet-to-lymphocyte ratio (PLR). We examined multicollinearity using variance inflation factors (VIFs). Model calibration and overall fit were evaluated using the Hosmer–Lemeshow goodness-of-fit test and pseudo-*R*^2^ indices (McFadden, Cox–Snell, and Nagelkerke). We inspected standardized residuals and influence statistics to assess major violations of logistic regression assumptions. Statistical significance was set at *p* < 0.05.

Analyses and plots were performed using IBM SPSS Statistics for Windows version 26 (Chicago IL. USA), RStudio 2023.03.0 + 386 “Cherry Blossom” for Windows.

## Results

### Patients' characteristics

We analyzed 83 patients who underwent Hartmann reversal during the study window. Among the 25 patients (30%) experienced a postoperative complication. Reintervention was the most frequent event (*n* = 13; 52%). Anastomotic leakage occurred in (*n* = 9; 36%). Additionally, 96% of patients with SF experienced other postoperative adverse events (Supplementary Table S1).

Baseline characteristics were comparable between groups. Median age was 49 years (IQR: 41–60) and 57% were male. Sex and age distribution did not differ between surgical success and surgical failure. Body mass index showed a similar pattern across groups. The main original indications for colostomy were complicated diverticular disease in 33 patients (40%), tumor in 6 patients (7.2%), and ballistic trauma in 8 patients (9.6%). There were no significant differences in indication profiles between outcomes (Table 1).

**Table 1:** Population characteristics according to surgical success.

Variable	Overall *N* = 83^a^	No *N* = 25^a^	Yes *N* = 58^a^	*p*-value^b^
Sex				0.258
Female	36/83 (43%)	8/25 (32%)	28/58 (48%)	
Male	47/83 (57%)	17/25 (68%)	30/58 (52%)	
Age (years)	49 (41–60)	49 (40–58)	49 (42–61)	0.528
BMI	25.80 (24.40–27.46)	25.90 (24.40–27.50)	25.75 (24.40–27.40)	0.956
Colostomy Cause				0.550
Ballistic trauma	8/83 (9.6%)	4/25 (16%)	4/58 (6.9%)	
Complicated Appendicitis	2/83 (2.4%)	1/25 (4.0%)	1/58 (1.7%)	
Complicated Diverticular Disease	33/83 (40%)	8/25 (32%)	25/58 (43%)	
Incidental disruption	14/83 (17%)	5/25 (20%)	9/58 (16%)	
Other	7/83 (8.4%)	3/25 (12%)	4/58 (6.9%)	
Strangulated hernia	2/83 (2.4%)	1/25 (4.0%)	1/58 (1.7%)	
Trauma	6/83 (7.2%)	1/25 (4.0%)	5/58 (8.6%)	
Tumor	6/83 (7.2%)	0/25 (0%)	6/58 (10%)	
Volvulus	5/83 (6.0%)	2/25 (8.0%)	3/58 (5.2%)	

a *n*/*N* (%); Median (Q1–Q3).

b Pearson's Chi-squared test; Wilcoxon rank sum test; Wilcoxon rank sum exact test.

Laparoscopic HR was performed in 52 patients (63%). Open reversal was performed in 26 patients (31%). The rest of the reported cases started as minimal invasion but were converted to an open surgical approach and conversion occurred in 6%. Surgical approach did not differ between groups (*p* *=* 0.766). The median surgical time was 184 min (IQR: 160–240; *p* *=* 0.073). Estimated blood loss was similar between groups. (*p* = 0.141). However, postoperative hospital stay was significantly longer in the patients with SF (8 days, IQR: 7–11) compared to the patients with SS (6 days, IQR: 5–8), (*p* = 0.001) (Table 2).

**Table 2:** Surgical characteristics according to hartmann reversal success.

Variable	Overall *N* = 83^a^	No *N* = 25^a^	Yes *N* = 58^a^	*p*-value^b^
Surgical approach				0.766
Conversion	5/83 (6.0%)	1/25 (4.0%)	4/58 (6.9%)	
Laparoscopic	52/83 (63%)	15/25 (60%)	37/58 (64%)	
Open	26/83 (31%)	9/25 (36%)	17/58 (29%)	
Surgical time (min)	184 (160–240)	210 (175–265)	180 (147–235)	0.073
Blood loss	100 (50–200)	100 (100–300)	100 (50–200)	0.123
Days of stay	7.00 (6.00–9.00)	8.00 (7.00–11.00)	6.00 (6.00–8.00)	0.001

a *n*/*N* (%); Median (Q1–Q3).

b Pearson's Chi-squared test; Wilcoxon rank sum test; Wilcoxon rank sum exact test.

#### Biochemical markers and CRP kinetics

Median total neutrophil count did not differ between groups [5.60 × 10^9^/L (3.30–8.13) vs. 4.49 × 10^9^/L (3.37–5.53); *p* = 0.290]. By contrast, the unsuccessful cohort showed a markedly lower total lymphocyte count [1.46 × 10^9^/L (1.20–1.97) vs. 1.81 × 10^9^/L (1.58–2.40); *p* = 0.025] (Supplementary Table S2). Platelet counts and serum albumin were comparable (*p* = 0.350 and 0.525, respectively).

Inflammatory dynamics differed over time. On POD1, CRP was slightly lower in the SF group [78 mg/L (57–86) vs. 97 mg/L (58–135); *p* = 0.049]; however, by day 3 the pattern reversed, with higher CRP in unsuccessful patients [240 mg/L (140–288) vs. 154 mg/L (92–185); *p* = 0.003]. The absolute ΔCRP magnified this divergence: unsuccessful patients demonstrated a median rise of 136 mg/L [53–218] compared with 56 mg/L [1–98] in the SF group (*p* < 0.001).

Composite inflammatory ratios echoed these findings. Although the neutrophil-to-lymphocyte ratio (NLR) trended higher in the unsuccessful cohort [3.75 (1.77–4.75) vs. 2.25 (1.59–2.97)], the difference did not reach significance (*p* = 0.100). In contrast, the platelet-to-lymphocyte ratio (PLR) was significantly elevated in SF cases [154 (126–208) vs. 124 (92–158); *p* = 0.012].

### Logistic regression analysis

In univariable analysis, higher ΔCRP [0.989; (95% CI: 0. 982–0.995); *p* ≤ 0.001] and lower PLR [0.987; (95% CI: 0.974–0.996); *p* = 0.019] were associated with a lower probability of surgical success, whereas BMI, surgical time, intraoperative bleeding, albumin, and NLR were not significantly associated (Table 3). In the multivariable model including ΔCRP, NLR, and PLR, ΔCRP remained independently associated with surgical success (OR: 0.987; 95% CI 0.977–0.995; *p* = 0.003), while NLR and PLR were no longer statistically significant (Table 3). VIF values for all covariates were below 2, indicating no evidence of problematic multicollinearity (Supplementary Table S3) The final multivariable logistic regression model showed acceptable calibration (Hosmer–Lemeshow *p* = 0.093) and good overall fit, with McFadden, Cox–Snell, and Nagelkerke pseudo-*R*^2^ values of 0.41, 0.41, and 0.57, respectively.

**Table 3:** Univariable and multivariable analysis.

Predictor	Univariable OR (95% CI)	*p*-value	Multivariable OR (95% CI)	*p*-value
BMI	1.040 (0.857–1.260)	0.719		
Surgical time (min)	0.997 (0.990–1.000)	0.227		
Intraoperative bleed	0.998 (0.995–1.000)	0.162		
Albumin	0.847 (0.313–2.060)	0.724		
ΔCRP	0.989 (0.982–0.995)	**<0** **.** **001**	0.987 (0.977–0.995)	**0** **.** **003**
NLR	0.840 (0.665–1.020)	0.095	1.06 (0.768–1.54)	0.738
PLR	0.987 (0.974–0.996)	0.019	0.984 (0.963–1.00)	0.117

Bolded values indicate statistically significant results (p ≤ 0.05).

### Diagnostic performance

**Figure 2:**
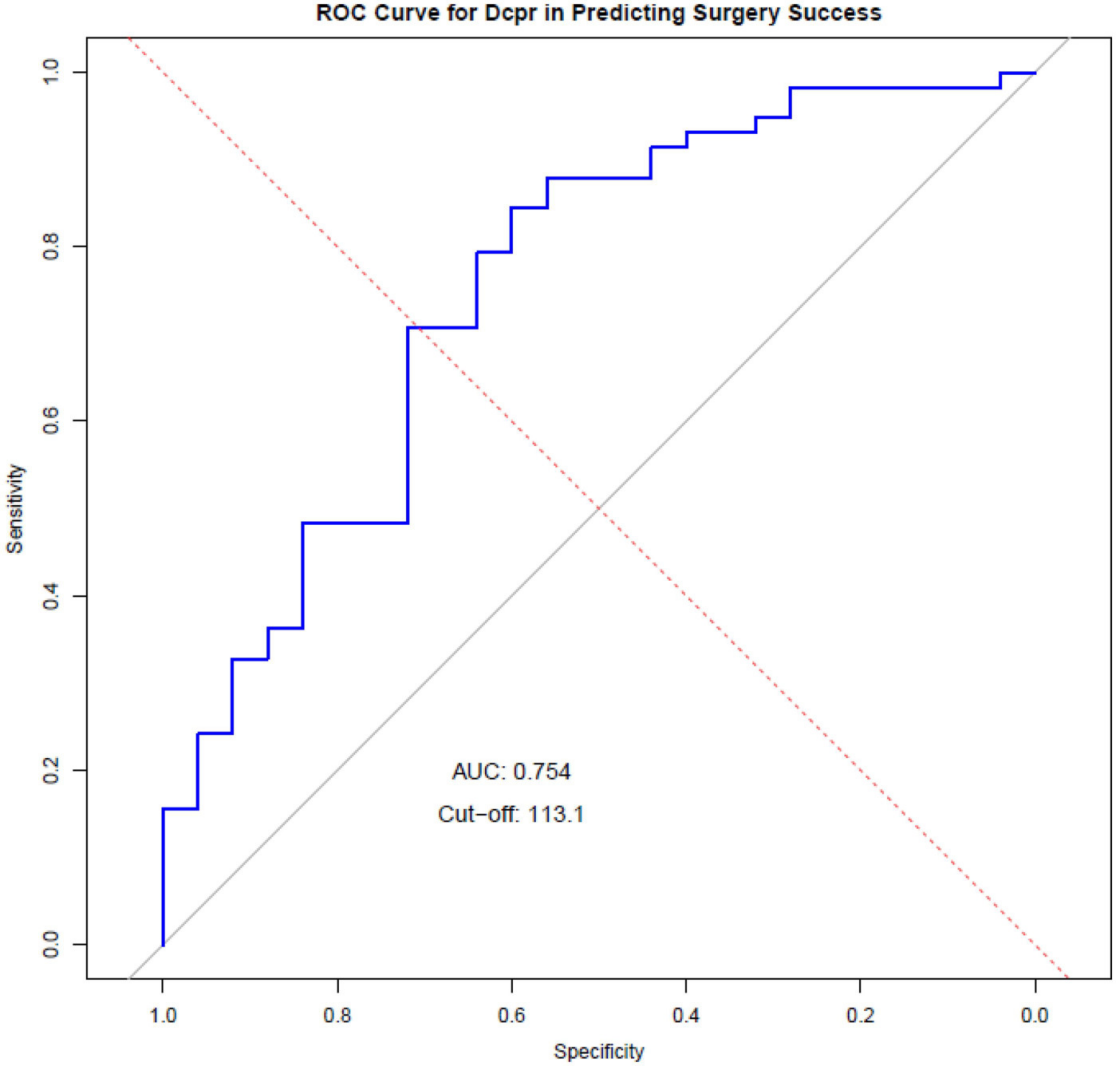
ROC analysis of CRP for predicting surgical failure. ROC curve sowing the discrimination of CRP for surgical failure. The marked point indicates the selected threshold (Th = 113.1). The area under the curve (AUC) was 0.754 (95% CI, 0.634-0.873; *p* < 0.001).

We evaluated the ability of ΔCRP to predict surgery success using ROC analysis. In Figure 2, the AUC is 0.754 (95% CI: 0.634–0.873), which reflects moderate discrimination. The SE is 0.061, and the AUC differs from 0.5 on the DeLong test with *p* = 3.07 × 10^−5^. The Youden rule selected a threshold of 113.1, giving sensitivity 84.5% and specificity 60.0% with a 40.0% false-positive rate. The Closest Top Left criterion suggested 84.0, yielding sensitivity 70.7% and specificity 72.0% with a 28.0% false-positive rate. At the Youden threshold, PPV was 83.1% and NPV was 62.5% in a sample where success prevalence was 69.9%. The corresponding likelihood ratios were 2.11 for LR+ and 0.26 for LR−, indicating that higher ΔCRP values increase the probability of success while lower values decrease it, and that results should be interpreted with clinical context.

In bootstrap internal validation, the mean AUC was 0.755 (95% CI of 0.631–0.867), consistent with the reported AUC. The mean optimal ΔCRP cut-off across bootstrap samples was 106.9 mg/L (95% CI: 48.5–169.6), indicating that the discriminatory region lies approximately within the 100–120 mg/L range rather than at a single exact value. In a sensitivity analysis of thresholds around the apparent Youden cut-off (113.1 mg/L), sensitivity ranged from 0.76 to 0.88 and specificity from 0.52 to 0.64, while PPV remained stable at ∼0.81–0.83 and NPV at ∼0.53–0.65, supporting the robustness of the model's performance in this range.

## Discussion

HR remains a challenging surgical procedure with substantial morbidity rates and variable postoperative outcomes. Identifying early, objective predictors of surgical success is crucial for optimizing patient selection, guiding perioperative management, and improving recovery. In this study, we found that ΔCRP between postoperative days 1 and 3 were significantly associated with surgical success, with a greater reduction in CRP levels correlating with lower complication rates and shorter hospital stays. These findings suggest that CRP kinetics, rather than absolute CRP values, provide a valuable early indicator of postoperative recovery in HR patients. By establishing a specific ΔCRP threshold (113.1 mg/L) for predicting surgical success, this study offers a practical and clinically relevant tool for postoperative risk stratification. These results reinforce the growing body of evidence supporting CRP as a key biomarker in colorectal and abdominal surgery and highlight its potential role in enhancing postoperative monitoring protocols and clinical decision-making.

These findings are consistent with prior research on the role of CRP in predicting postoperative complications, particularly in major abdominal and colorectal surgeries. Previous studies have identified elevated CRP levels as an early marker of anastomotic leakage, surgical site infections, and overall postoperative morbidity (16–18). However, while CRP kinetics have been explored in colorectal resections and general abdominal surgeries, their predictive value in the specific context of HR procedures has remained unclear. Given the high morbidity associated with HR (3, 5), our study provides new insights by demonstrating that ΔCRP may serve as a simple yet powerful biomarker for early risk stratification.

CRP rises over the first 24–48 h after major abdominal surgery and declines thereafter in uncomplicated recoveries. Small differences in the timing of “POD1” sampling can therefore shift early medians without reflecting a true difference in clinical trajectory. The more stable signal is the evolution from POD1 to POD3: patients with failure showed persistently elevated or rising CRP toward POD3, whereas successes tended to plateau or fall, which is consistent with expected postoperative kinetics. As a result, ΔCRP outperformed single-timepoint CRP in discriminating outcomes.

CRP is a well-established acute-phase reactant synthesized in response to systemic inflammation, infection, and tissue injury (9). While single postoperative CRP measurements have been widely used to assess surgical recovery, our study underscores the greater predictive value of CRP trends over time rather than absolute values at a single time point. This aligns with prior research suggesting that CRP trajectories offer superior accuracy in identifying patients at risk for postoperative complications (13, 19).

A key finding of our study is that a ΔCRP cutoff value of 113.1 mg/L was associated with significantly increased odds of surgical success, as demonstrated by the AUC. This suggests that patients with a greater reduction in CRP levels by postoperative day 3 are more likely to have an uncomplicated recovery, whereas those with persistently high CRP levels warrant closer monitoring and potential early intervention. The integration of CRP kinetics into routine postoperative monitoring protocols could enable clinicians to identify at-risk patients before clinical deterioration occurs, facilitating earlier imaging, targeted antibiotic therapy, and timely interventions (12, 20).

Several studies have explored the utility of CRP in predicting complications after colorectal surgery, but consensus on the optimal timing and cutoff values remains inconsistent. Some research has suggested that CRP levels exceeding 140–160 mg/L on postoperative day 3 are indicative of an increased risk for complications such as anastomotic leakage (9, 10, 19). However, our study refines this threshold in the HR population. This suggests that patients with a failure to decrease CRP below this threshold should be considered for enhanced postoperative surveillance.

### Strengths and limitations

This study reflects real practice in a public tertiary center where Hartmann reversal is performed regularly. The cohort included consecutive patients within a defined window, which reduces selection bias and mirrors the case-mix seen by multidisciplinary teams. The exposure of interest was simple and available in routine care. CRP was measured on postoperative day 1 and postoperative day 3, which are standard points in many pathways and capture the early inflammatory arc. Using the change between these two time points provides an interpretable signal that clinicians can apply at the bedside without extra cost or specialized assays. The outcome definition focused on events that matter to patients and services. Absence of major complications, no reoperation, and discharge within the expected recovery period are tangible targets for teams planning postoperative care. The analysis used familiar summary measures and widely understood tests, which supports reproducibility and clinical uptake. Taken together, these features increase feasibility and support near term translation of the findings into decision support after HR.

Our single-center retrospective design and relatively small sample size (*n* = 83) limit causal inference, increase the risk of residual confounding, and may restrict generalizability because thresholds for intervention, postoperative surveillance, and discharge can differ across institutions. The sample size was modest, which affects precision of estimates and the stability of subgroup effects. The ΔCRP cut-off was derived and internally explored using bootstrap resampling and sensitivity analyses, which suggest a robust discriminatory region within the 100–120 mg/L range. However, these procedures were performed in a single-center retrospective cohort, and external validation in independent populations is still required before adopting specific decision thresholds in routine practice. Although we excluded patients with overt infection unrelated to surgery and those on chronic immunosuppressive therapy, possibly adding selection bias, we did not systematically capture minor preoperative infections or short-term perioperative steroid use, which may still influence CRP kinetics and contribute to unmeasured confounding. This raises the risk of overfitting and underscores the need for external validation in diverse settings. Timing of blood draws, perioperative fluids, analgesic regimens, and antibiotics can influence CRP values. Although measurements were obtained on postoperative day 1 and postoperative day 3, variation in the exact clock time may still introduce measurement bias and noise into ΔCRP estimates. Only CRP was evaluated. Other inflammatory markers and composite clinical scores were not assessed, so potential incremental value remains unknown. Long term outcomes were not captured, and the definition of surgical success included discharge within a five day period. Discharge practices and bed availability may influence this component and could vary by center or season. Finally, exclusion of cases with missing CRP on both time points may introduce selection bias toward patients with more complete monitoring. These constraints should temper interpretation and guide the design of prospective multicenter studies that test predefined thresholds and assess clinical impact.

### Implications for practice

ΔCRP is practical for routine use after HR because CRP is already drawn on postoperative day 1 and day 3 in many pathways. Calculating the difference and recording it in the daily note can support bedside judgment without new resources. A small or falling ΔCRP aligns with an uncomplicated course and can reinforce standard mobilization, diet advancement, and discharge planning within ERAS protocols (8, 21). A large or rising ΔCRP should prompt caution with closer observation, early imaging when indicated, and a focused search for infection or leak (16–18, 22). Lines and drains should be reviewed, fluid balance reassessed, and antibiotics verified. The threshold observed in this cohort was about 113 mg/L and should be used as a guide rather than a rule, interpreted with the clinical picture and local capacity (23). Teams can integrate ΔCRP into handovers and track outcomes over time to refine local cut points and workflows. The aim is earlier recognition of deterioration and safer, timely discharge after HR.

### Future perspectives

Future research should focus on validating the ΔCRP threshold in prospective, multicenter trials to enhance its generalizability and clinical applicability. Additionally, integrating CRP kinetics into machine learning-based risk prediction models could improve real-time patient monitoring and early complication detection. Further studies should also assess the utility of additional inflammatory biomarkers, such as procalcitonin or interleukin-6, to determine whether a multimarker approach enhances predictive accuracy. Moreover, investigating the impact of perioperative interventions, including ERAS protocols and immunonutrition, on CRP kinetics and surgical outcomes may provide insights into modifiable factors that could improve postoperative recovery and reduce complication rates.

## Conclusion

By establishing ΔCRP as a valuable predictor of surgical success following HR procedures, this study provides a simple, cost-effective, and clinically relevant tool for early risk stratification. Patients with persistently elevated CRP levels were more likely to develop postoperative complications, emphasizing the role of CRP kinetics in guiding postoperative care decisions. The integration of CRP trends into clinical protocols and ERAS pathways could improve patient outcomes, reduce complication rates, and optimize hospital resource utilization. Further prospective validation and refinement of predictive models will be necessary to fully harness the clinical utility of ΔCRP in surgical practice.

## Data Availability

The raw data supporting the conclusions of this article will be made available by the authors, without undue reservation.
