# Supplementary material for: Early CRP kinetics predict surgical outcomes in patients with Hartmann reversal
Source: Front Surg. 2026 Feb 2;13:1732382. doi: 10.3389/fsurg.2026.1732382 (PMC12907330; doi:10.3389/fsurg.2026.1732382)
Supplement: Supplementary file 1 [file Table1.docx]

Table S1. Complications across sample

| **Variable** | **Overall**  N = 83^1^ | **Complications**  N = 25^1^ |  |  |  |
| --- | --- | --- | --- | --- | --- |
| **Reintervention** | 13 / 83 (16%) | 13 / 25 (52%) |  |  |  |
| **Anastomotic leakage** | 9 / 83 (11%) | 9 / 25 (36%) |  |  |  |
| **Rehospitalization** | 6 / 83 (7.2%) | 6 / 25 (24%) |  |  |  |
| **Other complications** | 24 / 83 (29%) | 24 / 25 (96%) |  |  |  |
| ^1^n / N (%). | | | | | |
| ^2^Pearson's Chi-squared test. | | | | | |

Table S2. Biochemical results among samples according to surgical success

| **Variable** | **Overall**  N = 83^1^ | **No**  N = 25^1^ | **Yes**  N = 58^1^ | **p-value**^2^ |
| --- | --- | --- | --- | --- |
| **Total neutrophils** | 4.65  (3.30-6.12) | 5.60  (3.30-8.13) | 4.49  (3.37-5.53) | 0.290 |
| **Total lymphocytes** | 1.80  (1.44-2.18) | 1.46  (1.20-1.97) | 1.81  (1.58-2.40) | **0.025** |
| **Platelets** | 235  (202-283) | 250  (219-309) | 235  (201-267) | 0.350 |
| **Albumin** | 4.41 (4.10-4.73) | 4.49 (4.17-4.78) | 4.41 (4.10-4.72) | 0.525 |
| **CRP in Day 1** | 86 (57-129) | 78 (57-86) | 97 (58-135) | **0.049** |
| **CRP in Day 3** | 167 (116-234) | 240 (140-288) | 154 (92-185) | **0.003** |
| **∆CRP** | 74 (13-123) | 136 (53-218) | 56 (1-98) | **<0.001** |
| **NLR** | 2.41 (1.67-4.11) | 3.75 (1.77-4.75) | 2.25 (1.59-2.97) | 0.100 |
| **PLR** | 136 (104-175) | 154 (126-208) | 124 (92-158) | **0.012** |
| ^1^Median (Q1-Q3). | | | | |
| ^2^Wilcoxon rank sum test. | | | | |

Table S3. Variance inflation factors candidate multivariable model

| **Variable** | **VIF** | **High VIF** |
| --- | --- | --- |
| **BMI** | 1.15060847 | No |
| **Surgical time (min)** | 1.15819622 | No |
| **Intraoperative bleed** | 1.22338213 | No |
| **Albumin** | 1.24271754 | No |
| **ΔCRP** | 1.14003289 | No |
| **NLR** | 1.60648192 | No |
| **PLR** | 1.70831952 | No |
| VIF: variance inflation factor. Values ≥5 were considered indicative of potentially problematic multicollinearity | | |
